# Sustainable financing of European non-governmental organizations (NGOs)

**DOI:** 10.1093/eurpub/ckab121

**Published:** 2021-08-12

**Authors:** Milka Sokolović, Dineke Zeegers Paget

**Affiliations:** 1 European Public Health Alliance (EPHA), Brussels, Belgium; 2 European Public Health Association (EUPHA), Utrecht, Netherlands

With the call from the EU President for a European Health Union, health has finally obtained its rightful place in the list of European priorities. An obvious reason is the COVID-19 pandemic, stressing the importance of strong, resilient health systems and the need for a considerably increased health budget at EU level. The adoption of the EU4Health Programme 2021–2027, with the largest health budget ever, represents an important milestone in ensuring that health remains a policy priority at EU level, providing a foundation to support the health and wellbeing of everyone in Europe. This ambitious funding mechanism, however, harbours a paradox that threatens to take European health a step back, by limiting support for one of its major actors—the (public) health civil society.

Over the years, evidence—such as the EU’s midterm evaluation of the 3rd Health Programme 2014–2020^1^ and the WHO/European Health Observatory ‘Civil Society and Health’ book^2^—has shown that non-governmental organizations (NGOs) are an effective partner. In previous EU Health Programmes, NGOs have brought value through sharing best practice and exchanging knowledge across Europe, making collaboration with civil society an established mechanism of health governance and governance for health.

In partnering with NGOs to achieve the Health Programmes’ objectives, operating grants have been instrumental in supporting their long-term sustainability and capacity. NGOs typically directly add value by connecting and building bridges: by listening and amplifying the voices in their communities and those of their members and partners, incorporating them into the policy discussion, and by partnering in policy implementation and running initiatives on the ground on national and regional level. These actions require a stable organization, and operating grants have proved a worthy enterprise, offering stable and predictable funding to NGOs, and enhanced administrative and financial benefits for all concerned. It is our direct experience that the operating grants remain a valued mechanism in the EU health policymaking to ensure adequate funding for NGOs and enable their role in the health debate, making sure that the voice of the most vulnerable is heard.

It is therefore a paradox that the added value of European health umbrella organizations is set to be declining instead of increasing in the EU4Health programme, by phasing out operating grants and introducing a (fragmented), project-based, ‘action grant’ approach. The action grants are being considered as an alternative, but they currently do not guarantee sustainable, predictable funding for NGOs. They come with no evidence-based justification for the decision, with little clarity on the grants’ scope, size and process, and with an extremely narrow window for the civil society to adjust to the change. This leaves organizations reliant on short-term, project-specific funding, making their day-to-day operations unsustainable and threatening their added value. And it forces them to explore alternative funding, such as relying on private sector, which can jeopardize their independence and therefore their valuable watchdog role.

## The key challenges: independence, continuity, level playing field

A study commissioned by the European Parliament identifies the challenges that NGOs face in the EU, with their independence from private funding and vested interests as a major one.^3^ It also points out that the EU institutional evidence shows that due to developments over the past 5 years, conditions have worsened for NGO actors, and especially for critical organizations that operate across the EU.^4^

A recent study on ‘The future evolution of civil society in the European Union by 2030’ concludes that the EU has the right tools, including funding instruments, that can and should be used to support NGOs in successfully navigating through the identified societal challenges, working with them to establish a better European process of governance, and helping them enhance participatory democracy by and beyond 2030.^5^ Both the challenges and opportunities have been acknowledged by other Directorates General of the European Commission through offering a continuous operating support to European organizations active in fields such as social policies and environment, which leaves health NGOs in a discriminated position.

Health in EU would only benefit from a clear, publicly stated, long-term commitment to involving and supporting health civil society organizations and recognizing their role in the EU4Health Programme. Discontinued structural funding puts the health NGOs, especially those operating at European level and covering a broad range of (public) health topics, at the verge of survival, risking discontinuity of their existence, or the lack of legibility for the upcoming funding, may they turn to the private finding to bridge the gap period.

## Disclaimer

Both the European Public Health Alliance and the European Public Health Association have received funding through an EU operating grant.

*Conflicts of interest*: None declared.
